# Cross-cohort single-nucleotide-variant profiling of gut microbiota suggests a novel gut-health assessment approach

**DOI:** 10.1128/msystems.00828-23

**Published:** 2023-10-31

**Authors:** Chenchen Ma, Yufeng Zhang, Shuaiming Jiang, Fei Teng, Shi Huang, Jiachao Zhang

**Affiliations:** 1 Key Laboratory of Food Nutrition and Functional Food of Hainan Province, School of Food Science and Engineering, Hainan University, Haikou, China; 2 School of Medicine, Southern University of Science and Technology, Shenzhen, China; 3 Faculty of Dentistry, The University of Hong Kong, Hong Kong SAR, China; 4 Qingdao Stomatological Hospital Affiliated to Qingdao University, Qingdao, China; 5 One Health Institute, Hainan University, Haikou, Hainan, China; Northern Arizona University, Flagstaff, Arizona, USA

**Keywords:** single-nucleotide variation, gut microbiome, variant bias, short-chain fatty acids, gut microbiome health index, SNV rate

## Abstract

Adaptive evolutionary changes can precede the ecological changes in the gut microbial communities under constant host selection pressure, yet their association with host diseased status was underexplored. Explore shared disease-associated single-nucleotide variants (SNVs) in gut microbes spanning diverse human diseases. We performed a meta-analysis of 1,711 gut metagenomic samples from 16 case-control studies spanning 12 human diseases and included an additional 446-member cohort for validation. Overall, healthy individuals carried more mutated resident gut microbes, and more SNVs, mainly involving the short-chain fatty acids (SCFAs) producing bacteria. Furthermore, the widespread differences in base variant bias of gut microbes were observed between healthy and nonhealthy subjects suggesting divergent gut microbial evolutionary directions under different host medical conditions. We further found that nonsynonymous SNVs can lead to the loss of function of four SCFA-production genes in nonhealthy populations, among which two genes (i.e., *ack* and *scpC*) from *Faecalibacterium prausnitzii* C and *Bacteroides stercoris,* respectively, were externally validated. Subsequently, we developed a novel gut microbiome health index based on the SNV rate of all mutated strains, classifying host health states with 74.23% accuracy, and validated with high accuracy (AUROC = 69.28%). Our study highlights the importance of employing the genetic variability in gut microbiome to characterize the gut microbial adaptation that can also predict human chronic diseases.

**IMPORTANCE:**

Most studies focused much on the change in abundance and often failed to explain the microbiome variation related to disease conditions, Herein, we argue that microbial genetic changes can precede the ecological changes associated with the host physiological changes and, thus, would offer a new information layer from metagenomic data for predictive modeling of diseases. Interestingly, we preliminarily found a few genetic biomarkers on SCFA production can cover most chronic diseases involved in the meta-analysis. In the future, it is of both scientific and clinical significance to further explore the dynamic interactions between adaptive evolution and ecology of gut microbiota associated with host health status.

## INTRODUCTION

Gut microbes consistently evolve by gaining the adaptive SNVs and structural variants (SV) in response to the gut selection pressures derived from host physiological changes, dietary changes, antibiotic use, and probiotics interventions (1–5). In the last decade, many SNVs and SVs of gut microbiota have been reported, which underlie the adaptive evolution of microbial functional genes that can be associated with phenotypic changes in both microbes themselves and hosts (6, 7). However, more studies are still required to understand the population-level genetic processes of gut microbiota under a broad spectrum of host health conditions (8).

Recently, a range of metagenomics studies assessed the genetic variation of host gut microbes and reported characteristic SNVs associated with a few chronic diseases. A set of SNVs in specific gut microbial residents have been associated with host health status, such as *Faecalibacterium prausnitzii* and *Eubacterium rectale* [colorectal cancer (CRC) (9), liver cirrhosis (LC) (10), graves’ disease (GD) (11), Inflammatory bowel disease (IBD) (12)], *Bacteroides vulgatus* [tuberculosis:TB (13), GD (11)]. These bacteria usually carried a large number of SNVs, and their SNV profiles were different between healthy and nonhealthy groups. Many studies also implied that a small number of genetic variants or even a single SNV in the microbial genome can significantly alter the pathogenic behavior of gut bacteria and affect host health. For example, T2D patients often possessed SNVs enriched on the glycosyl hydrolases gene of *Bacteroides coprocola*, which was recognized as an important gut-microbiome-derived therapeutic target for T2D (14). Furthermore, it is evident that a single SNP (G84E) of *Escherichia coli* can disturb gut lysophospholipid homeostasis and induce host inflammation by epithelial barrier disruption (15). Nevertheless, the consensus microbial SNV signatures associated with a wide range of human chronic diseases have never been attempted. Furthermore, no studies have systematically assessed if these widespread SNVs in the gut microbiota from multiple diseases link to any core health-related functions of hosts. For example, short-chain fatty acids (SCFAs) produced by gut bacterial fermentation of dietary fibers are widely considered key bacterial metabolites regulating host immune response or anti-inflammatory factors (16, 17). Associations between fecal SCFA level and host health have been widely found in a variety of diseases, including COVID-19 (18), type 2 diabetes (T2D) (19), colorectal cancer (20), Crohn’s disease (CD), ulcerative colitis (UC) (21), Parkinson (22), polycystic ovary syndrome (PCOS) (23), diabetic nephropathy (24), encephalitis (25), etc. We hypothesized that a metagenomic meta-analysis of disease-related data sets would identify cross-disease microbial SNV signatures which can also link to core microbial functional genes modulating the host health.

The large population size in the meta-analysis is required to consolidate the SNV findings and further uncover the potential impact of SNV in intestinal microbes on the nonhealthy hosts. Currently, the number of public metagenomes in repositories is growing exponentially (26). The meta-analysis of publicly accessible shotgun metagenomic data is an economical and powerful way (27) to identify universal gut-microbiota-derived biomarkers for multiple chronic diseases. Importantly, going beyond conventional abundance data, profiling the genetic variability in gut microbes can expand our understanding of the evolutionary and ecological processes in the gut underlying the disease development. However, it is still technically challenging to compare the SNV profiles across samples by the rigorous consideration of variation in sequencing depth and coverage among samples.

To address the above challenges, we systematically studied the SNVs in gut resident microbial strains that can associate with host selection pressures under 12 human medical conditions. We attempted to test if the evolutionary directions for individual gut microbes differ between healthy and nonhealthy subjects. Most previous studies reported a decrease in the relative abundance of SCFA-producing gut strains in nonhealthy subjects. More importantly, we found that SCFA production in nonhealthy individual intestinal tract was inactivated due to adaptive variants in related genes more prone to turn codons into terminators, further suppressing the overall concentration of SCFAs in the GI tract. Finally, we established the “Gut Microbiome Health Index” (GMHI) using SNV profiles and benchmarked its prediction performance against that derived from the species-level abundance profiles conventionally used in most past studies. Notably, SNV profiles of individual gut microbes exhibited a strong predictive power in evaluating the health status of human hosts.

## MATERIALS AND METHODS

### Metagenomic data set collection and curation

To perform a comprehensive metagenomics meta-analysis for identifying the consensus in genetic variations in gut microbes associated with human diseases, we extensively searched for publicly accessible metagenomic data sets using a range of key words (e.g., “shotgun,” “gut microbiome,” “intestinal microbiome,” “metagenomic,” “metagenome,” “whole genome sequence”) in PubMed and ISI Web of Science (as of October 2021). Metagenomic studies related to dietary, drug, or antibiotic interventions were excluded. We also require that the cohort must include both healthy and nonhealthy individuals, which ensures that the cohorts are comparable with each other. Specially, if a study collected nonhealthy samples from multiple time points, we included only the first or baseline samples. Finally, we pinpointed 15 studies for our meta-analysis, a total of 1,711 metagenomic samples from 919 nonhealthy and 792 healthy individuals were included, spanning 12 host phenotypes (Cov19, Covid-19; SCZ, Schizophrenia; SF, stone formers; AS, atherosclerosis; PCOS, polycystic ovary syndrome; GD, graves' disease; T2D, type 2 diabetes; CRC, colorectal cancer; BC, breast cancer; UC: ulcerative colitis; CD, Crohn’s disease; LC, liver cirrhosis), representing a comprehensive set of human health conditions. Publicly available raw sequences (.fastq) and corresponding metadata were downloaded from NCBI SRA database. The detailed technical information on each study, such as shotgun metagenomics sequencing platform, average sequencing depth, target read lengths, and other profiles, was shown in Table 1. Notably, UC and CD cohorts share the same healthy control group. Two validation cohorts (AVCD, atherosclerotic cardiovascular disease; TB, tuberculosis) were collected using the consistent technical standards as we used for discovery cohorts.

**TABLE 1:** Fecal metagenomic studies included in this meta-analysis

Study^*a*^	Country	Nonhealthy sample size	Healthy sample size	Total	Sequencing platform	Average sequencing target depth	Target read length	Sequencing data
Cov19	China	15	15	30	Illumina NextSeq 550	4.39 GB	150 bp	PRJNA624223
SCZ	China	90	81	171	Illumina HiSeq X	10.94 GB	150 bp	PRJEB29127
SF	Italy	5	5	10	Illumina NextSeq 500	1.57 GB	150 bp	PRJNA418941
AS	China	97	114	211	Illumina HiSeq 2000	3.73 GB	100 bp	PRJNA375935
PCOS	China	14	14	28	Illumina HiSeq 4000	6.22 GB	150 bp	PRJNA549764
	China	50	43	93	Illumina HiSeq 2500	8.78 GB	150 bp	PRJNA530971
GD	China	102	62	164	lllumina HiSeq 2500	8.07 GB	100 bp	PRJNA602729, PRJNA602731, PRJNA602732, PRJNA638403, PRJNA638404, PRJNA638405
T2D	China	71	74	145	Illumina GAIIx and HiSeq 2000	2.51 GB	175 bp	PRJNA422434
CRC	China	8	12	20	lllumina HiSeq 2500	6.45 GB	150 bp	PRJNA663646
	Japan	40	40	80	lllumina HiSeq 2500	6.42 GB	150 bp	DRA006684
	Italy	32	28	60	lllumina HiSeq 2500	3.89 GB	100 bp	SRP136711
	Austria	46	63	109	lllumina HiSeq 2000	4.84 GB	100 bp	ERP008729
BC	China	62	71	133	ION_TORRENT	10.94 GB	150 bp	PRJNA718520
UC	American, Holland	76	56	220	lllumina HiSeq 2500	4.01 GB	101 bp	PRJNA400072
CD	American, Holland	88				4.34 GB		
LC	China	123	114	237	Illumina HiSeq 2000	1.74 GB	100 bp	PRJEB6337
		919	792	1,711				

^*a*^ Cov19, Covid-19; SCZ, schizophrenia; SF, stone formers; AS, atherosclerosis; PCOS, polycystic ovary syndrome; GD, graves' disease; T2D, type 2 diabetes; CRC, colorectal cancer; BC, breast cancer; UC, ulcerative colitis; CD, Crohn's disease; LC, liver cirrhosis.

### Quality control of the raw metagenomic data

Sratoolkit 2.10.7 software (https://github.com/ncbi/sra-tools) was performed to separate raw sra files into paired or single fastq files. The raw reads were trimmed using Sickle (https://github.com/najoshi/sickle) and subsequently aligned to the host genome (GRCh38) to remove the host DNA fragments using Bowtie2 (28) with default settings.

### Species taxonomic profiling

First, we identified microbial species and estimated their relative abundances in each stool sample using MetaPhlAn 2.8 using default parameters based on the database (mpa_v29_CHOCOPhlAn_201901) (29). Next, alpha diversity was calculated based on the species abundance profiles.

### SNV calling for resident gut microbiota

We mainly conducted this analysis using inStrain. First, Bowtie2 was applied to map our reads with a default reference genome of inStrain including 204,938 genomes, 4,644 representative strain genomes to create bam files. Then, each sam file was converted to a sorted and indexed .bam file using samtools (30). Lastly, inStrain was used to call SNVs; analyze the sequencing coverage and breadth of mutated genomes, scaffolds, genes; estimate the species-level nucleotide diversity; etc. with default parameters (31). Notably, the minimum read-to-genome ANI (--min_read_ani) was set to be 95% by default, where all reads are expected to have an actual 95% ANI to species representative genomes (i.e., at the strain level, https://instrain.readthedocs.io/en/latest/index.html). The pN/pS of a gene and variant bias of genome were calculated at positions where at least two alleles present rather than in relation to the reference genome. We next attempted to calculate the relative frequency of SNVs on a given genome (i.e., SNV rate) that can compare with other genomes. The raw SNV count is not an ideal measurement as the sequencing depth and breadth can vary substantially among mutated genomes. Therefore, we derived a new normalized SNV count metric, SNV rate, as follows:


(1) $$SNV\;rate=\frac{SNV\;number}{g\_len\times breadth\_minCov}$$

SNV number indicates the number of SNVs called from a mutated genome in a metagenomic sample. g_len indicates the genome length of this mutated genome. breadth_minCov, a typical inStrain output, indicates the percentage of bases in a scaffold/genome that has at least min_cov coverage. Particularly, it refers to the percentage of bases that have a nucleotide diversity value and meet the minimum sequencing depth that allowed us to call SNVs. Therefore, SNV rate was calculated as the percentage of bases that can call single-nucleotide variants by all SNV-callable bases in a microbial genome.

We next estimate the relative sequence abundance for mutated genomes using the relative number of individual reads that pass the selecting pairing filter which can be found in the inStrain output “mapping_info.tsv.” Gff profiles for each genome are required to call SNVs and can be accessed at https://doi.org/10.5281/zenodo.4441269.

### The normalized SNV count by sequencing depth

We investigated the sequencing depth for all 1,711 samples using seqkit 2.1.0 (32). Of note, our previous study has shown the strong correlation between raw number of SNVs and sequencing depth (3). Certainly, we want to reconfirm this relationship with simulated data and determine the relationship between other profiles (e.g., the number of mutated genomes) and the sequencing depth. To solve the above issue, first, we selected six samples from different cohorts (three healthy and three nonhealthy) from 1,711 samples, and their sequencing depths were slightly higher than 10G. Next, we apply seqtk 1.3 to extract sequencing data from fastq files randomly (https://github.com/lh3/seqtk). And then, we acquired simulated data from 1G to 10G (step size: 1G) with three times (seed = 11, 12, and 13). Our results suggested the particularly strong positive correlation between the number of mutated genomes and SNV and the sequencing depth (Supplementary file 1; Fig. S1, *R* = 0.988–0.999, *P* < 0.001). The simulation results in the range of 10G sequencing depth are applicable to most samples (Supplementary 1; Fig. S2). So, in this study, number of mutated genomes and SNV was normalized based on sequencing depth.

### Establish a GMHI based on SNV profiles and genome-level sequence abundances

GMHI based on species relative abundance was previously proposed to distinguish healthy from nonhealthy groups (33), which can be potentially developed as a new diagnostic tool for host health. Here, we sought to test if any improvement in prediction accuracy using the variability in genetic composition within each species to assess host health status as compared to genome-level abundance profiles. Therefore, we generate three feature tables for building up a predictive index for health status (i.e., GMHI): (i) the sequence abundance profiles for all mutated genomes, (ii) SNV rate for all mutated genomes (Eq 1), and (iii) SNV count for all mutant genes producing SCFAs for each sample.

GMHI based on SNV rate of mutated genomes.

1. The health- and nonhealthy-enriched markers were identified for each feature table using multiple Wilcoxon rank-sum tests. The compositional data were central-log-ratio transformed prior to statistical tests. Each feature table was further filtered by healthy enriched markers (MH) and healthy depleted markers (MN), respectively (all markers can be found in https://github.com/HNUmcc/Meta_SNV_2157/tree/main/data).
2. In either MH or MN sub-table, we calculate Shannon diversity (Hs or Ns) and richness (Hr or Nr) based on markers for each sample.
3. We further calculated the median Hr (or Nr) from 1% of the top (bottom)-ranked samples from (Hp or Np).
4. We next calculated the “collective” sequence abundance, SNV rate of mutated genomes or SNV frequency of genes producing SCFAs for health-enriched (i.e., psi_H) or nonhealthy enriched markers (i.e., psi_N) for each metagenomic sample.

(2) $$psi\_H=\frac{Hr}{Hp}\times Hs$$

(3) $$psi\_N=\frac{Nr}{Np}\times Ns$$

5. Calculating a GMHI for each sample,


(4) $$GMHI=log_{10}\left(\frac{psi\_H}{psi\_N}\right)$$

The necessary scripts and markers of three profiles can be found by https://github.com/HNUmcc/Meta_SNV_2157.

### Statistics analysis

The statistical analysis was performed using R software. The differential abundances of various profiles were tested with the Wilcoxon rank-sum test with fdr adjust if needed, and the significant difference was considered at a nominal level of *P* < 0.05. Alpha diversity analysis was performed “picante” and “vegan” package. Beta diversity analysis was conducted using “vegan,” “plyr,” and “ggExtra” package; PCoA based on Bray-Curtis and Euclidean dissimilarity matrix was used to visualize the sample clustering based on gut microbial or gut mutated genomes composition; Adonis analysis was conducted using the vegan package; and the permuted *P* value was obtained by 999 pervariants. The package “ggplot” and “gghalves” were used to generate boxplot, violin plot, density plot, and fitted curve. The heatmap was constructed using the “pheatmap” package.

## RESULTS

### Data set collection and meta-analysis workflow

**Fig 1:**
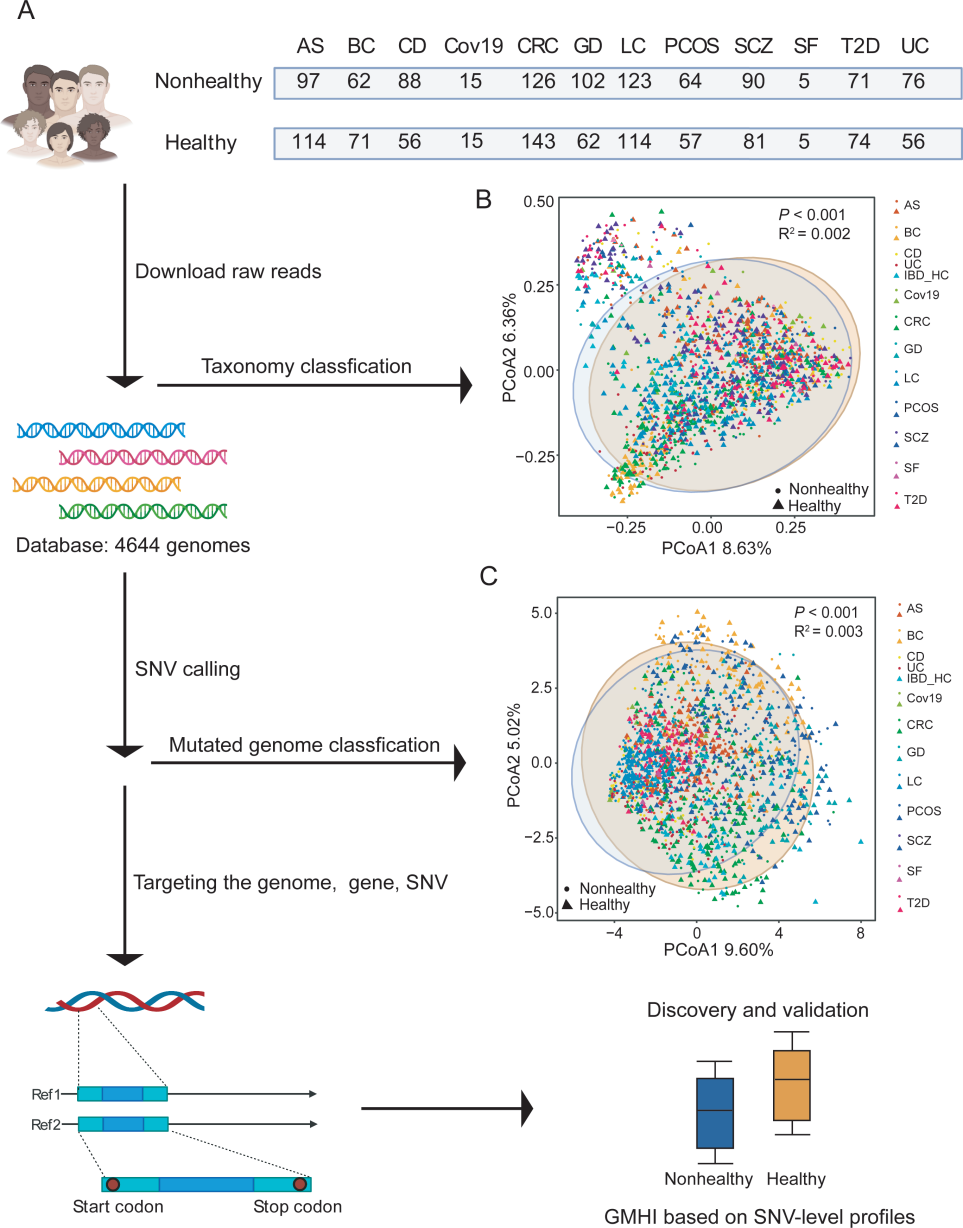
Data set integration and meta-analysis workflow. (**A**) In this study, 1,711 metagenomic samples from 16 microbiome studies (including 919 healthy and 792 nonhealthy individuals) were integrated into our meta-analysis for calling, detection, and profiling of genetic variation in the gut microbiome. Each of 16 cohorts in this study has both healthy and nonhealthy individuals. First, comparisons between different health status were limited to one nonhealth phenotype and, more importantly, global comparisons. Next, specific mutated genomes, genes, and SNVs were mentioned to assess the impact of SNVs on health status. (**B**) Principal coordinate analysis (PCoA) plot based on Bray-Curtis dissimilarity showed the between-sample difference in gut microbial composition between nonhealthy (circle, *n* = 919) and healthy (triangle, *n* = 792) groups. (**C**) PCoA based on Euclidean distance showed a significant difference in the diversity of mutated genomes in the gut microbiota between nonhealthy and healthy groups. For each PCoA plot, each dot corresponds to a sample, and dot’s shape corresponds to the health status, and dot’s color corresponds to the disease phenotype. The ellipse corresponds to 95% confidence region.

In this meta-analysis, we collected 1,711 samples from 16 metagenomics studies spanning 12 host phenotypes, including 919 nonhealthy and 792 healthy human individuals (Fig. 1A; Supplementary file 2 and Table S1). Notably, subjects were categorized into “healthy” or “nonhealthy” according to the original studies, while overweight and obesity alone individuals were not classified as nonhealthy group. Expectedly, we observed beta-diversity differences based on species-level fecal microbial abundance between healthy and nonhealthy groups using Bray-Curtis dissimilarity (*R* = 0.002, *P* < 0.001, Adonis, Fig. 1B, 95% confidence regions) based on normalized data, consistent with the past results in most studies.

Subsequently, we sought to explore the disease-associated genetic variation in gut microbiota by mapping metagenomics reads against a comprehensive set of gut microbial reference genomes. If we identified any SNVs from a microbial reference genome, this “mutated genome” or SNV-carrying microbial strain will be further analyzed to characterize its SNV profile and test if it can associate with disease status (Supplementary file 2 and Table S2). We identified a total of non-redundant 2,740 mutated genomes in 1,711 samples. Here, to compare genomic variation across samples, we focused on 233 (8.5%) genomes with high prevalence (≥10%). A meaningful comparison of genomic variation between samples requires both breadth and depth of sequencing for each genome (6). In this study, the sequencing coverage of each SNV was required to be 5, minimally, which could cover genomes (average coverage of 158 genomes more than 5, 67.81%) (Supplementary file 1 and Fig. S1). Interestingly, we next observed the beta-diversity difference based on the composition of SNV-carrying strains (*N* = 2,740) between healthy and nonhealthy groups (*R* = 0.003, *P* < 0.001, Adonis, Fig. 1C, 95% confidence region) based on Euclidean dissimilarity matrix. This suggested that host medical conditions can associate with not only population-level abundance changes but also genetic compositional changes in the gut microbiota. Next, we focused on specific strains, genes, and SNVs, showing the association between gut microbial SNVs and host health status.

### The healthy gut microbiome harbors a wider range of SNV-carrying strains

According to inStrain pipeline for analysis of co-occurring genome populations (see Materials and Methods), we obtained the comprehensive SNV profiles of gut microbiota for both healthy and nonhealthy cohorts. Of note, in this study, the number of strains and SNV count was normalized based on metagenomic sequencing depth (see “Materials and Methods,” Supplementary file 1 and Fig. S2).

**Fig 2:**
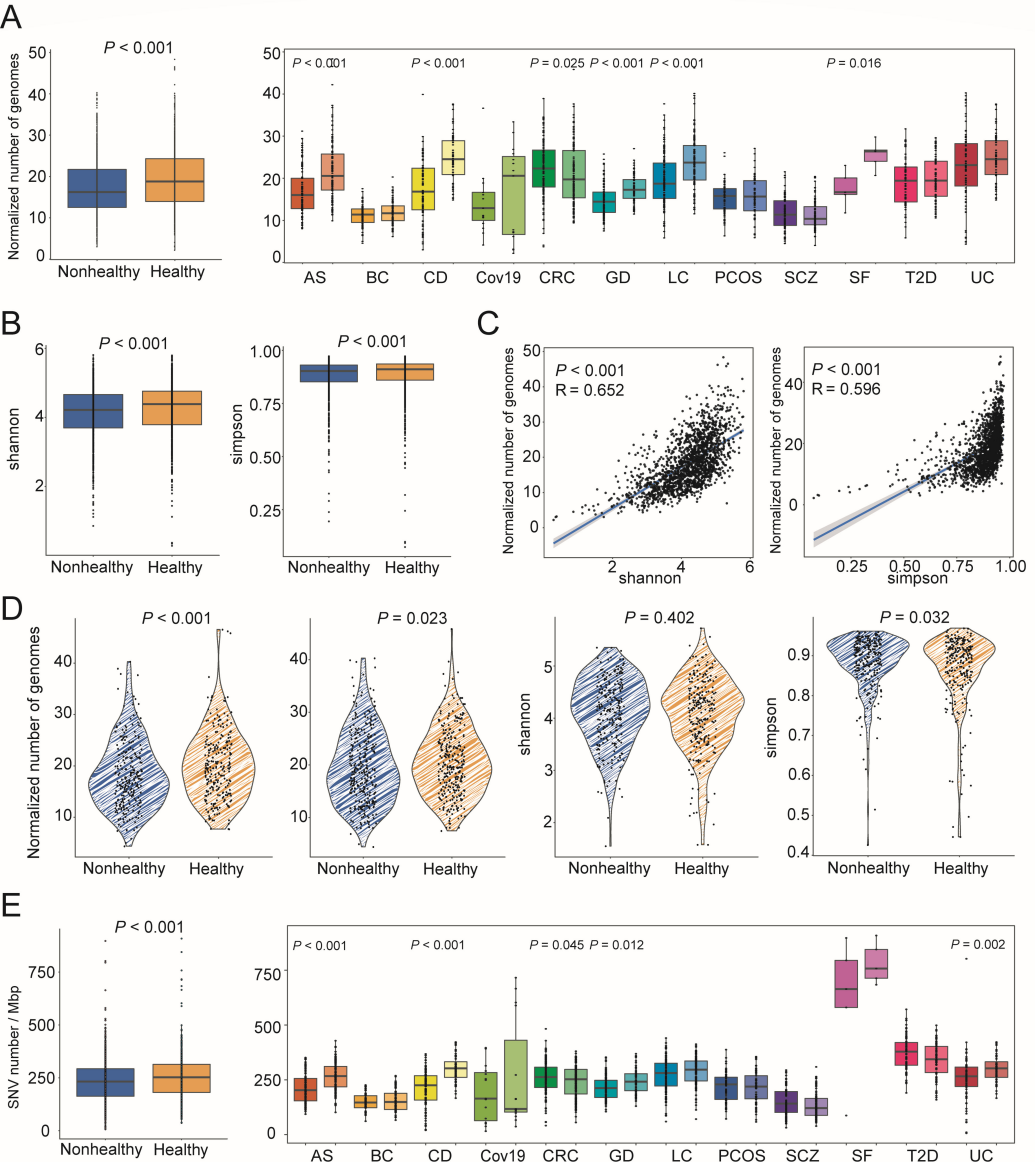
Normalized evaluation and comparisons of number of mutated genomes and SNVs. (**A**) The boxplot shows the comparison of normalized number of mutated genomes in the whole data set (*N* = 1,711) and for each disease phenotype (Wilcoxon rank sum test). (**B**) Comparison of alpha diversity (Shannon and Simpson index) based on gut microbial profiles at the species level. (**C**) The strong correlations between number of mutated genomes and Shannon index (Spearman correlation). (**D**) Matched individuals with the same alpha diversity from healthy and nonhealthy groups were used to compare alpha diversity, and individuals with the same number of mutated genomes were also compared with the number of mutated genomes (Wilcoxon sign rank test). (**E**) Comparison of the normalized number of SNV of gut microbes between healthy and nonhealthy individuals in whole data set (*N* = 1,711) and each disease phenotype by Boxplot (Wilcoxon rank sum test). The *P* values were corrected with fdr method, and the significant difference was considered at a nominal level of *P < 0.05, **P < 0.01, and ***P < 0.001. For all boxplots, the nonhealthy group was on the left and the healthy group was on the right.

We first explored the difference in the richness of mutated strains, and the total number of SNVs between healthy and diseased states. First, we observed that healthy subjects (19.48 ± 7.38) had more mutated strains than diseased subjects (17.39 ± 6.84) (Fig. 2A; Supplementary file 2 and Table S3, *P* < 0.001), suggesting a higher strain-level diversity in the healthy gut microbiota. Respectively, such a difference pattern was also found in a total of six independent cohorts corresponding to six diseases, including AS (16.92 ± 5.64 vs 21.93 ± 7.23, *P* < 0.001, average ± SD), CD (17.33 ± 6.87 vs 24.76 ± 5.77, *P* < 0.001), GD (14.78 ± 3.75 vs 17.61 ± 3.37, *P* < 0.001), LC (19.51 ± 6.01 vs 23.77 ± 6.51, *P* < 0.001), and SF (17.56 ± 3.81 vs 25.51 ± 3.0, *P* = 0.016). In contrast, CRC individuals had a higher strain-level diversity than healthy control (22.26 ± 6.80 vs 20.73 ± 7.11, *P* = 0.025). There were no significant differences in other cohorts, including BC (11.10 ± 2.66 vs 11.83 ± 2.83), Cov19 (14.29 ± 7.41 vs 16.28 ± 10.84), PCOS (15.32 ± 3.87 vs 15.6 ± 14.67), SCZ (11.56 ± 3.71 vs 11.29 ± 3.45), T2D (19.13 ± 5.61 vs 19.78 ± 5.18), and UC (22.91 ± 8.62 vs 24.76 ± 5.77).

Most studies have reported differences in the species-level alpha diversity of healthy and nonhealthy cohorts. Here, our integrated analysis showed that alpha diversity (using either Shannon or Simpson index) is different between host disease states (Fig. 2B, *P* < 0.001, Wilcoxon rank-sum test). Intriguingly, the richness of mutated genomes significantly correlates with Shannon index (*R* = 0.652, *P* < 0.001) or Simpson (*R* = 0.596, *P* < 0.001) (Fig. 2C). One possibility is that lower intestinal microbial diversity leads to a smaller number of mutated genomes. To address these concerns, we matched healthy and nonhealthy patients with alpha diversity within 0.01 (Shannon index) or 0.001 (Simpson index) differences to compare their normalized number of mutated genomes (92.87% and 92.69% samples were included), and Shannon and Simpson index were 0.01 or 0.001 higher in nonhealthy group than in healthy. In such a manner, we found that when the alpha diversity is almost the same, the number of mutated genomes is different (Wilcoxon rank-signed test, *P* < 0.001 for samples with equalized Shannon index, and *P* = 0.023 for samples with equalized Simpson index). Therefore, in most cases, we believe that healthy individuals have higher strain-level diversity than nonhealthy individuals (Fig. 2D; Supplementary file 2 and Table S4). At the same time, we also matched individuals with <0.01 difference in the normalized number of mutated genomes and compared their alpha diversity (27.47% samples were included) between host groups. Interestingly, we still observed the disease-related difference in Simpson index at the species level (*P* = 0.032), where healthy group had less microbial species (Fig. 2D; Supplementary file 2 and Table S4).

Next, we estimated the SNV number per Mkb sequencing data for all mutated genomes in both healthy and nonhealthy gut microbiota, and demonstrated healthy individuals have more SNVs in total than nonhealthy individuals (23.44 ± 9.93 vs 25.20 ± 10.11, Fig. 2E, *P* < 0.001). Respectively, we validated such a difference in a total of four diseases/cohorts, including AS (212.85 ± 67.13 vs 264.80 ± 65.15, *P* < 0.001), CD (211.48 ± 81.11 vs 301.02 ± 61.23, *P* < 0.001), GD (213.46 ± 54.13 vs 241.71 ± 56.14, *P* = 0.012), and UC (257.86 ± 107.20 vs 301.02 ± 61.23, *P* = 0.002). By contrast, CRC individuals have more SNVs than healthy control (260.39 ± 75.34 vs 241.52 ± 71.09, *P* = 0.045). No significant differences were found in other cohorts, including BC (148.22 ± 40.47 vs 156.71 ± 49.76), Cov19 (190.14 ± 125.20 vs 260.43 ± 238.00), LC (270.74 ± 77.99 vs 289.17 ± 64.32), PCOS (214.99 ± 73.86 vs 216.58 ± 65.64), SCZ (148.39 ± 62.71 vs 135.49 ± 58.73), SF (604.81 ± 280.69 vs 781.80 ± 83.49), and T2D (371.49 ± 78.86 vs 339.52 ± 79.23). Given the genetic compositional difference found at such a higher level between disease states, SNV profiles on specific strains/mutated genomes need to be revealed further.

### Strain-level diversity associated with multiple host health status

**Fig 3:**
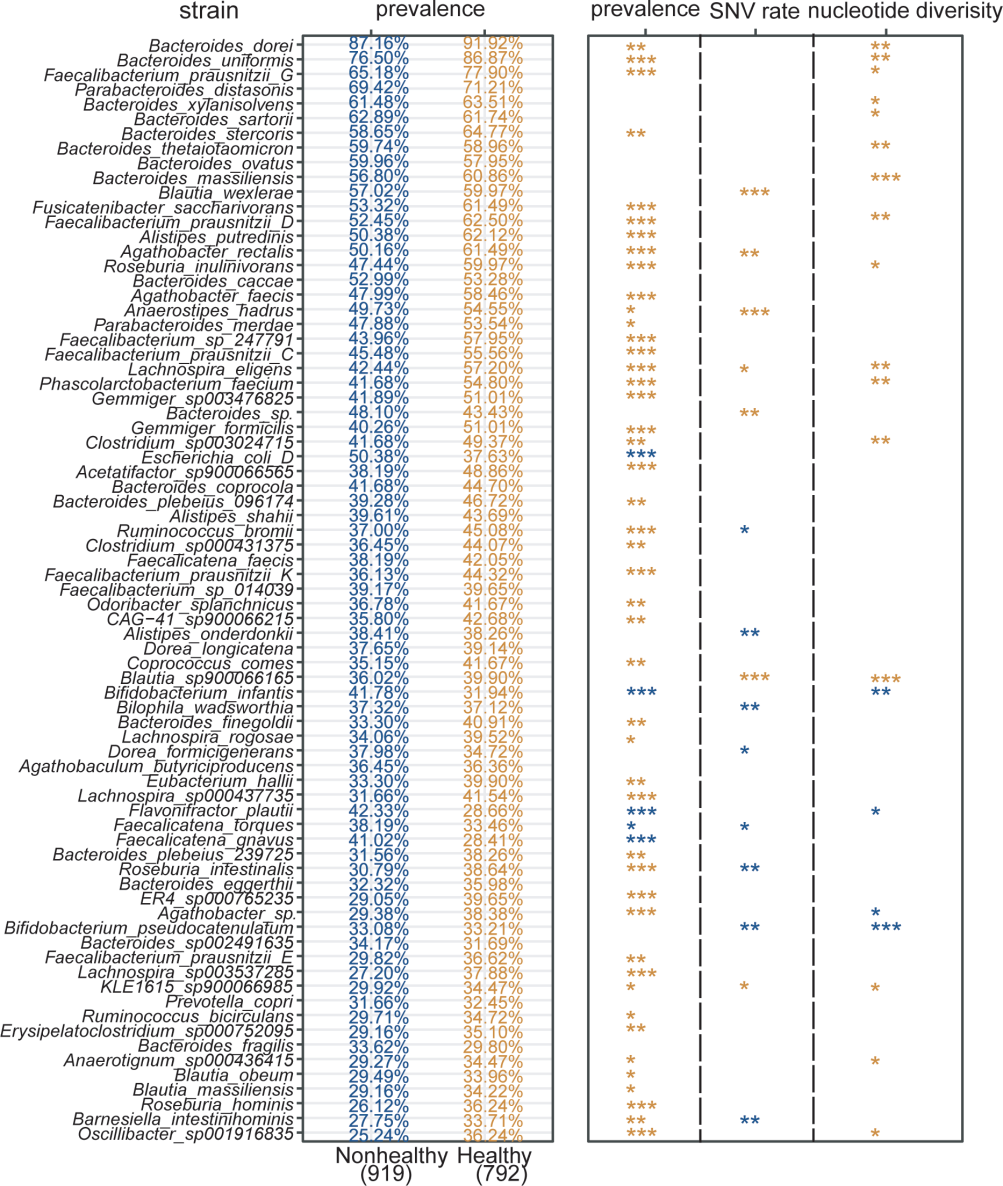
The overall strain-level diversity associated with host health status. A total of 75 mutated genomes were identified in >30% of 1,711 human individuals. The overall features [i.e., mutated genome frequency, SNV rate (see Materials and Methods), nucleotide diversity] for each of these 75 mutated strains, were compared between healthy and nonhealthy cohorts using Wilcoxon rank-sum test and Fisher’s exact test, and the significant difference was considered at a nominal level of *P* < 0.05. The *P* values were corrected with fdr method, and the significant difference was considered at a nominal level of *P < 0.05, **P < 0.01, and ***P < 0.001.

To further explore if disease states can also associate with the SNV profiles for individual microbial strains and other key evolutionary patterns for gut microbiota, we comprehensively analyzed all 75 strains with a variant frequency of more than 30% in the 1,711-member population (Fig. 3), and the corresponding genome ids of these strains can be found in Supplementary file 2 and Table S2. First, we compared the prevalence of these strains (Supplementary file 2 and Table S2), and the top four most prevalent strains in both healthy and unhealthy groups were *Bacteroides dorei* (nonhealthy: 87.16%, healthy: 91.92%, all: 89.36%), *Bacteroides uniformis* (nonhealthy: 76.50%, healthy: 86.87%, all: 81.30%), *Faecalibacterium prausnitzii* G (nonhealthy: 65.18%, healthy: 77.90%, all: 71.07%), and *Parabacteroides distasonis* (nonhealthy: 69.42%, healthy: 71.21%, all: 70.25%). Forty-six strains were more prevalent (46/75, 61.33%) in the healthy group, while five strains (5/75, 6.67%) were more prevalent in the nonhealthy group. At the species level, many strains from *Bacteroides* consistently have a high frequency of SNVs occurred in the healthy gut. At the strain level, *Faecalibacterium prausnitzii* G, *Faecalibacterium prausnitzii* D, *Faecalibacterium prausnitzii* C, *Faecalibacterium prausnitzii* K, and *Faecalibacterium prausnitzii* E also presented diverse patterns between healthy and nonhealthy population. Overall, the above results also confirm that the healthy group will have a wide range of variants in the genome (Fig. 2A).

We next investigated if SNV rate (see Materials and Methods, Eq 1) and nucleotide diversity of these gut microbial strains can associate with disease states. “SNV rate” is defined as the relative frequency of SNVs on a given genome that can compare with other genomes. Nucleotide diversity is a measurement of intra-population genetic diversity for a microbial species in the gut microbiota (Supplementary file 1 and Fig. S3). Clearly, the SNV rate and nucleotide diversity substantially varied among microbial species and even strains (e.g., *Faecalibacterium prausnitzii*). We identified 15 strains (out of 75 strains, 20%) that had a SNV-rate difference between disease states. Among these 15 strains, 8 strains (8/75, 10.67%) were higher in the nonhealthy group and 7 (7/75, 9.33%) strains were higher in the healthy group. Remarkably, 8 out of 15 strains (8/15, 53.33%) were mainly from Lachnospiraceae, including *Blautia wexlerae*, *Agathobacter rectalis*, Anaerostipes hadrus, *Lachnospira eligens*, *Blautia* sp900066165, *Faecalicatena torques*, *Roseburia intestinalis*, KLE1615 sp900066985, a typical group of bacteria that produce SCFA (i.e., acetate and butyrate). In contrast, *Bacteroides* were the main genus (6 strains, 6/20, 30%) with nucleotide diversity difference between disease states, including *Bacteroides dorei*, *Bacteroides uniformis*, *Bacteroides xylanisolvens*, *Bacteroides sartorii*, *Bacteroides thetaiotaomicron,* and *Bacteroides massiliensis*, and only three strains (3/75, 4%, 2 *Bifidobacterium*) were enriched in the nonhealthy group, while 17 (17/75, 22.67%) were enriched in the healthy group. Overall, these results confirmed that healthy individuals have more SNVs in their gut microbial genomes (Fig. 2E).

### Universal base variant bias associated with host health states

**Fig 4:**
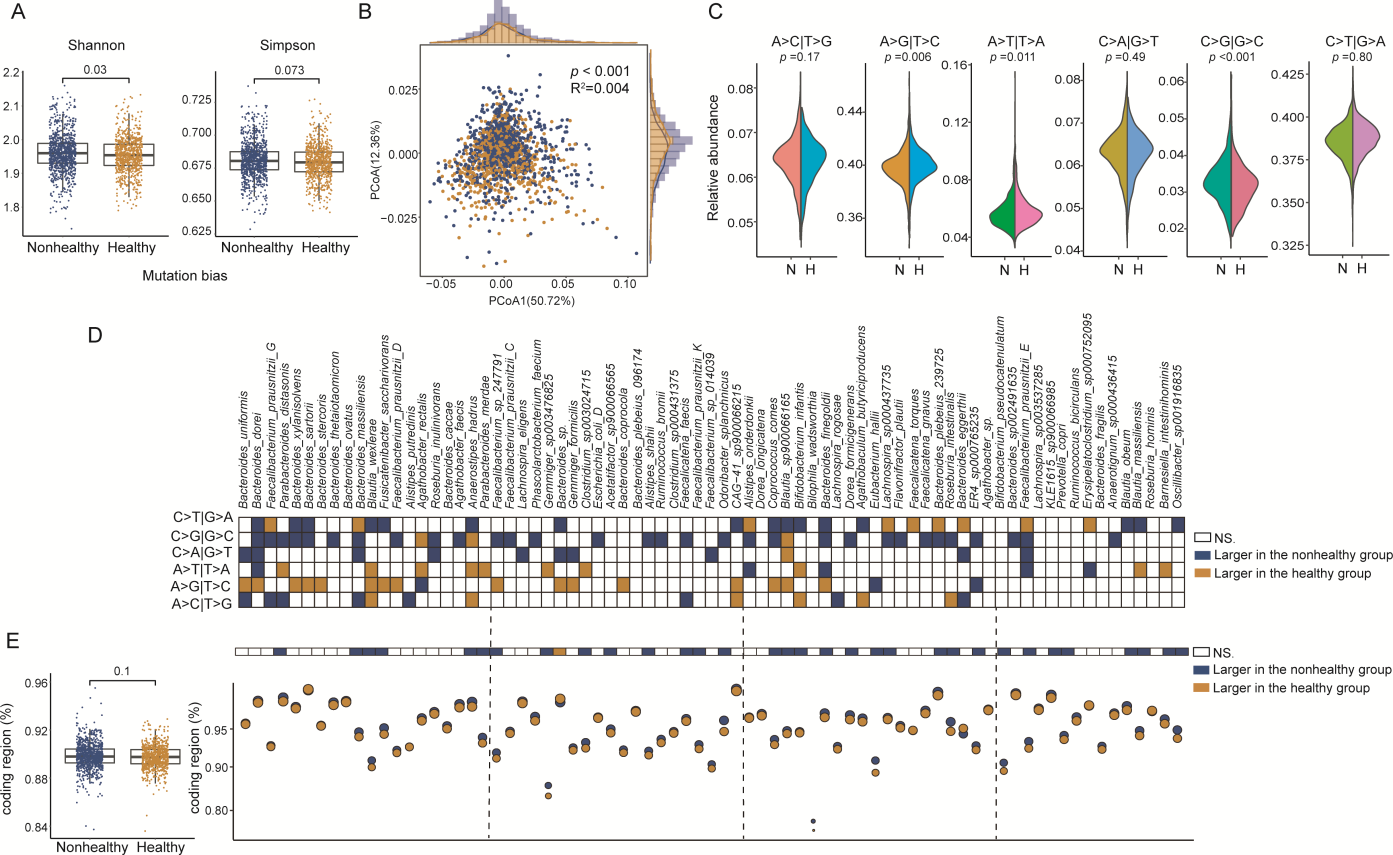
Universal base variant bias associated with host health status. (**A**) The comparison of Shannon index and Simpson index of variant-type profiles between healthy and nonhealthy groups (Wilcoxon rank-sum test). (**B**) The PCoA plot shows the between-sample difference based on Bray-Curtis dissimilarity of six-base variant-type profiles. Adonis was used to estimate the effect size of host health status on the variant types. The histograms show the distribution of healthy (orange) and nonhealthy (blue) samples along each axis. (**C**) The proportion of six-base variant types between healthy and nonhealthy groups was shown and compared in the half violin diagrams. N means nonhealthy group, and H means healthy group. (**D**) The relative abundance of six variant types of 75 mutated genomes was compared, and white mean no difference in base variant bias, orange mean higher in the healthy group, and blue mean higher in the nonhealthy group. (**E**) The proportion of SNVs located in the gene coding region was evaluated for the whole data set and for each of 75 strains. The *P* values were obtained from Wilcoxon rank-sum tests with fdr correction, and the significant difference was considered at a nominal level of *P* < 0.05.

Next, we narrowed down to the specific pattern of variant types at the single SNV level (i.e., nucleotide diversity) since the SNV rate and nucleotide diversity of 75 gut strains were associated with disease states. First, we profiled six variant types of all 1,771 samples (Supplementary file 2 and Table S5) and compared alpha diversity and beta diversity based on variant-type profiles across samples. Intriguingly, Shannon diversity for the variant types (*P* = 0.03) had significant differences between two groups (Fig. 4A). In addition, the variant-type composition of 75 gut microbial strains was primarily clustered by host disease states (*R*^2^ = 0.004, *P* < 0.001, PCoA1 = 50.72%, PCoA2 = 12.36%) (Fig. 4B). Specifically, we found that two variant types (A > G|T > C, *P* = 0.006 and A|T > T > A, *P* = 0.011, Wilcoxon rank-sum test) were enriched in the healthy group while one in the nonhealthy group (C > G|G > C, *P* < 0.001, Wilcoxon rank-sum test) (Fig. 4C; Supplementary file 2 and Table S5). We also showed and compared the variant types between host states for each strain (Supplementary file 2 and Table S6). Overall, we observed the diverse distribution patterns (percentage) of variant types among microbial strains (Fig. 4D). C > G|G > C in many strains tend to be significantly higher in nonhealthy people (31/75, 41.33%), while such variant type in only three strains were higher in the healthy group (3/75, 4%). In contrast, the A > G|T > C mutant type was more common in multiple strains in the healthy group (16/75, 21.33%).

We next compared the overall percentage of point variants occurring in the gene-coding region for all 75 strains, and no differences between disease states were found (89.84% vs 89.75%, *P* = 0.1, Fig. 4E). However, at the strain level, we observed the percentage of variants in gene-coding regions of 32 strains were significantly higher in the nonhealthy group than in the healthy group (32/75, 42.67%), while only three strains (*Bacteroides* sp.) were higher in the healthy group (3/75, 4%). This suggested that disease states can change SNV patterns in gene-coding regions of gut microbial strains.

### Strain-level codon variant bias in SCFA-production-involved genes

**Fig 5:**
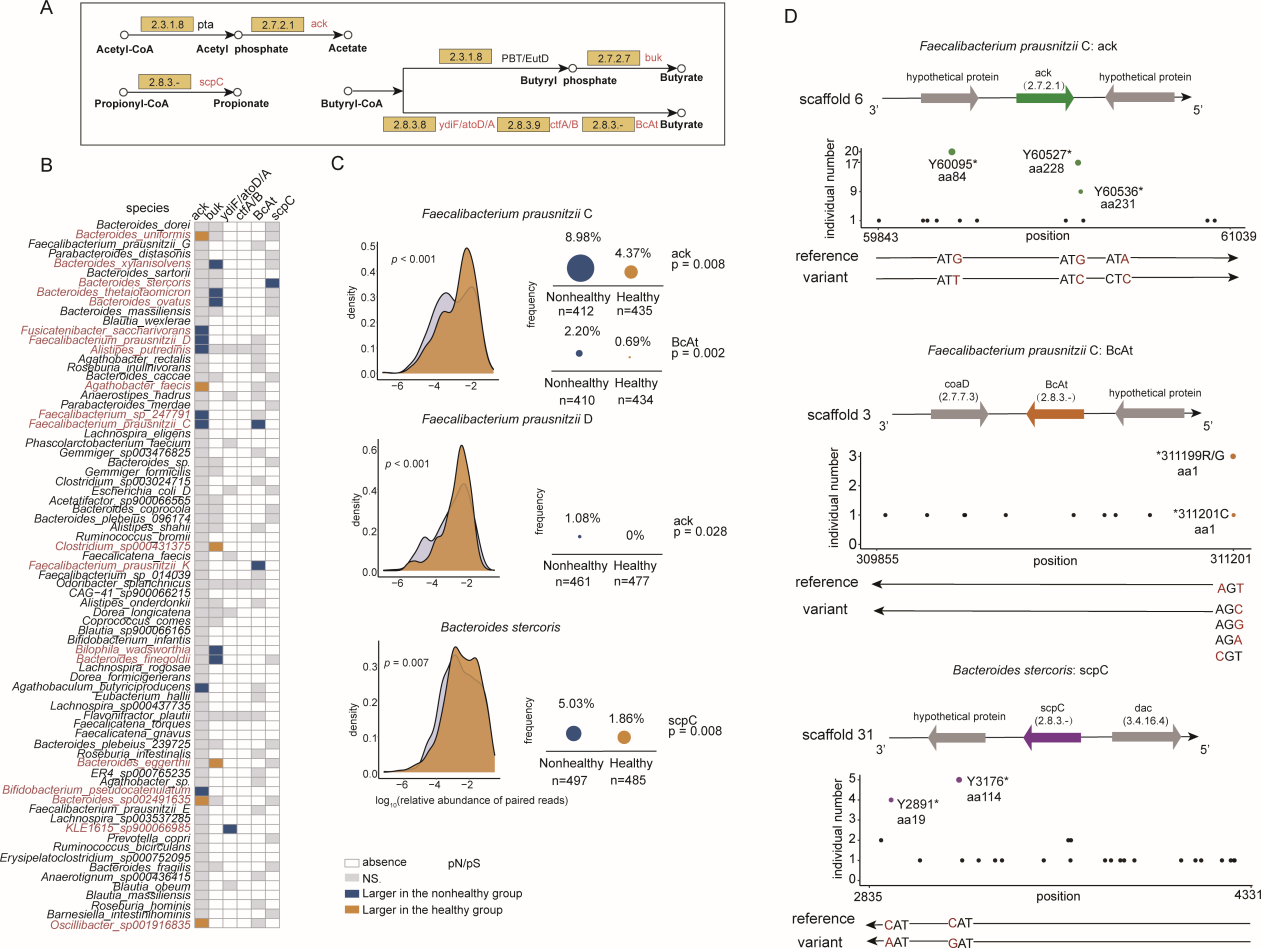
Strain-level codon variant bias in SCFA-production-involved genes. (**A**) The enzymes related to the production of three short-chain fatty acids (acetate, propionate, and butyrate) are shown. *ack*, acetate kinase; *scpC*, propionyl-CoA:succinate CoA transferase; *buk*, butyrate kinase; *ydiF*: acetate CoA-transferase YdiF; *atoD/A*, acetate CoA-transferase subunit alpha/beta; *ctfA/B*, butyrate--acetoacetate CoA-transferase subunit A/B; *BcAt*, butanoate coenzyme A-transferase. (**B**) The heatmap shows enrichment directionality of pN/pS (i.e., natural selection forces) for each of six SCFA-related gene families (including a total of 185 genes) encoded by 75 strains between healthy and nonhealthy groups. The pN/pS is the ratio of two rates: the rates of non-synonymous (pN) and synonymous (pS) SNVs. White cells indicate gene absence in a strain, gray cells mean no difference of pN/pS, orange cells mean larger pN/pS in the healthy group, and blue cells mean larger pN/pS in the nonhealthy group. (**C**) The SCFA genes of the three strains had codon variant bias, suggesting that SNV causes codon to become termination codon or the start codon is inactivated. The density plot displays the sequence relative abundance of the three strains in both healthy and nonhealthy groups. The between-group comparison was performed by using Wilcoxon rank-sum test. *N* represented the number of individuals with SNV on these genes. When the prevalence was calculated, the denominator was *N*, and the numerator was the number of individuals with codon variant bias. The *P* values from Fisher’s exact test and the significant difference were considered at a nominal level of *P* < 0.05. (**D**) Some SNVs were detected in at least three individuals, which causes codon to become termination codon or the start codon is inactivated. The horizontal axis represented the position of SNV, the vertical axis represented the number of individuals, and in addition, the scaffold, codon, reference genome, variant genome, and adjacent genes were also shown.

We next focused on key functional genes of specific gut strains and assessed potential functional changes in gut microbiota induced by microbial adaptive evolution under disease conditions. SCFA is widely considered to be closely related to human health, and SCFAs abundance deficiency has been observed in a variety of diseases, and the genetic evolution process of SCFA-related genes has not been reported so far. We then first counted the enzymes encoded by these strains that are related to SCFA production, including acetate kinase (*ack*, E.C. 2.7.2.1), propionyl-CoA:succinate CoA transferase (*scpC*, E.C. 2.8.3.-), butyrate kinase (*buk*, E.C. 2.7.7.7), acetate CoA-transferase YdiF (*ydiF*, E.C. 2.8.3.8), acetate CoA-transferase subunit alpha/beta (*atoD/A*, E.C. 2.8.3.8), butyrate—acetoacetate CoA-transferase subunit A/B (*ctfA/B*, E.C.2.8.3.9), and butanoate coenzyme A-transferase (*BcAt*, E.C. 2.8.3.-) (Fig. 5A).

We next presented the SNV profile of 75 strains encoding the above SCFA-related enzymes (Fig. 5B). We observed that almost all strains encode *ack* gene (74/75, 98.67%), involved in the acetate production. 74.67% strains have at least one metabolic pathway associated with butyrate production, and six strains have at least two pathways. In addition, *Bacteroides* spp. are the main producers of propionate (16/21, 76.19%). Then, we compared whether the pN/pS values of these genes were different between healthy and nonhealthy cohorts to assess the size of intestinal selection pressure. Its pN/pS ratio in four strains was higher in the healthy group, while seven strains were higher in the nonhealthy group. Differential intestinal selection pressures related to diseases drove the production of butyrate by related genes in 10 strains. Only *scpC* gene of *Bacteroides stercoris* had a difference in pN/pS, and the nonhealthy group was higher than the healthy group. Overall, the adaptive evolutionary patterns of SCFA-associated genes in different strains are diverse due to host health status.

Next, we explored if and how these SNVs specifically modulated the production of SCFAs. Here, we compared the frequency that a SNV on SCFA-related gene that causes codon to become termination codon or the start codon was inactivated (Fig. 5C). Intriguingly, those SNVs were mainly found in three strains (*Faecalibacterium prausnitzii* C, *Faecalibacterium prausnitzii* D, and *Bacteroides stercoris*). The frequency of SNV-derived codon changes in the nonhealthy group was significantly higher than that in the healthy group due to the disease-related selection pressure (Supplementary file 2 and Table S7). Respectively, two genes of *Faecalibacterium prausnitzii* C possessed codon variant bias affecting initiation and termination codon, including *ack* (8.98% vs 4.37%, *P* = 0.008) and *BcAt* (2.20% vs 0.69%, *P* = 0.002). In addition, codon variant bias also appeared in *ack* gene of *Faecalibacterium prausnitzii* D (1.08% vs 0%, *P* = 0.028) and *scpC* gene of *Bacteroides stercoris* (5.03% vs 1.86%, *P* = 0.008). Therefore, the adaptive variants that occurred in the above strains in the nonhealthy group would loss of function for SCFA-production genes. Although the SCFA genes of some six strains were subjected to greater intestinal selection pressure in the healthy group, it did not cause an increase in the above codon variant types (Supplementary file 1 and Fig. S4). We also compared the relative abundance of the four strains aforementioned between host states and found that the three strains were more abundant in the healthy group, including *Faecalibacterium prausnitzii* C (0.56% vs 0.61%, *P* < 0.001), *Faecalibacterium prausnitzii* D (0.51% vs 0.43%, *P* < 0.001), and *Bacteroides stercoris* (2.43% vs 2.56%, *P* = 0.007). Finally, we reported potentially common codon variants in gut microbial strains (prevalent in >3 host individuals, Fig. 5D; Supplementary file 2 and Table S7) affecting SCFA production, including three amino acids of *Faecalibacterium prausnitzii* C *ack* gene, one amino acid of *Faecalibacterium prausnitzii* C *BcAt* gene, and two amino acids of *Bacteroides stercoris scpC* gene.

### GMHI based on SNV profiles indicates host health status

Gut Microbiome Health Index (GMHI) was proposed to develop for predicting disease presence (or absence) using species-level taxonomic abundance profiles derived from metagenomic sequencing data. Overall, microbial abundance essentially reflects the ecological changes related to disease states. Given our findings above, we hypothesized that SNV profile can also predict the host states, reflecting the degree and extent by which gut microbes adapt under multiple disease conditions.

**Fig 6:**
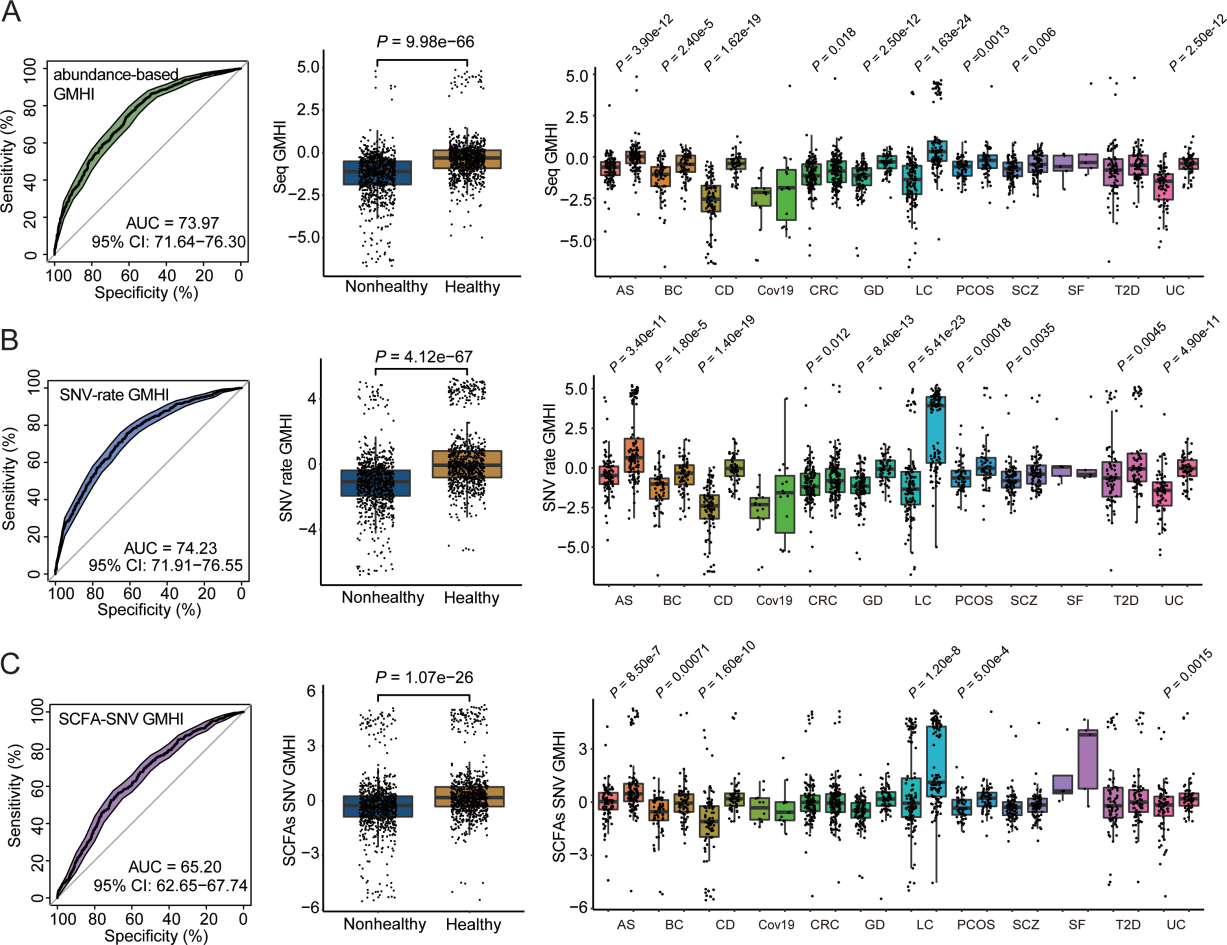
GMHI based on SNVs profiles indicates host health status. GMHI was calculated based on sequence abundance profiles of mutated genomes (i.e., abundance-based GMHI), SNV-rate profiles of mutated genomes (i.e., SNV-rate GMHI), and SNV profile of SCFA genes (i.e., SCFA-SNV GMHI), respectively. (**A**) The prediction performance of abundance-based GMHI (AUROC: 73.97%). (**B**) The prediction performance of the GMHI based on SNV rate profiles (AUROC: 74.23%) *P* = 4.12e−67, and 10 cohorts are applicable. (**C**) The predictive performance of the GMHI based on SNV profiles of SCFA genes (AUROC: 65.20%). The GMHI values were compared between healthy and nonhealthy groups using Wilcoxon rank-sum test. The significant difference was considered at a nominal level of *P* < 0.05. For all boxplots, the nonhealthy group was on the left and the healthy group was on the right.

First, to test and validate how well the abundance-based GMHI can predict the host states, sequence abundance profile of the mutated genomes was used to construct GMHI (abundance-based GMHI, see “Materials and Methods”). The relative sequence abundance of a total of non-redundant 2,740 strains was calculated for 1,711 metagenomics samples in the discovery cohorts and 446 samples in the validation cohort. Notably, we selected microbial strains differentially abundant in DNA sequence between diseased states as the biomarkers for calculating GMHI, which was slightly different from the previous study (33) (“Materials and Methods” and “Code availability”). Among 471 selected strain-level markers, 222 were enriched in the nonhealthy group, while 249 were enriched in the healthy group. We found that the overall prediction accuracy of the abundance-based GMHI was 73.97%, *P* = 9.98e−66, while this GMHI showed a significant difference between healthy and nonhealthy groups in nine cohorts (Fig. 6A). The result was similar to the reported accuracy of the original GMHI method (~70%) based on the taxonomic abundance of gut microbes at the species level (33).

We next explored if SNV rate, representing the scale of genetic variation for each microbial strain (Eq 1), can be used as an independent indicator to predict host disease states. Therefore, we calculated the SNV rate of each of non-redundant 2,740 strains in each sample, which formed a new SNV-rate feature table as compared to conventionally used abundance-based feature table. Next, we identified microbial strains that have a significant difference in the SNV rate between host states and used their SNV rate to construct a new GMHI for classifying disease states (see Materials and Methods). Among 470 selected markers, 214 were enriched in the nonhealthy group, while 256 were enriched in the healthy group. With the receiver operating characteristic curve (ROC), we further showed high prediction accuracy of this SNV-rate GMHI (AUROC = 74.23%, Wilcoxon rank-sum test, *P* = 4.12e−67). Its performance was slightly better than the strain-level abundance-based GMHI. Furthermore, we found this GMHI can distinguish host states in 10 independent cohorts (Fig. 6B).

We have shown that the variant frequency/bias of SCFA genes in gut microbes can potentially distinguish health states. Therefore, we next explored the feasibility of constructing GMHI based on the SNV profile in SCFA genes of 75 microbial gut strains. We collected 185 genes from nine gene families (*ack*, *buk*, *ydiF*, *atoD*, *atoA*, *ctfA*, *ctfB*, *BcAt*, and *scpC*) from 75 strains that carried adaptive SNVs. We first formed a count table for the SNVs (presence and absence) identified in the 185 genes. Then, we aggregated the SNV count to the genome level and calculated the relative frequency of SNV for SCFA genes in each genome in each sample (see “Materials and Methods,” Supplementary file 2 and Table S8). Among 48 selected markers, 11 were enriched in the nonhealthy group, while 37 were enriched in the healthy group. With the selected microbial SNV biomarkers, this SCFA GMHI showed that fairly good prediction accuracy (AUROC = 65.20%, Wilcoxon rank-sum test, *P* = 1.07e−26). Among 12 cohorts in this meta-analysis, the SCFA-based GMHI can be validated in six cohorts (Fig. 6C). Although this accuracy is not perfect, it strongly suggested that genetic variability in the SCFA genes alone can explain, at least partially, the microbiome difference between host health conditions.

### External validation of functional changes in SCFA production induced by SNVs and the predictive power of GMHIs

**Fig 7:**
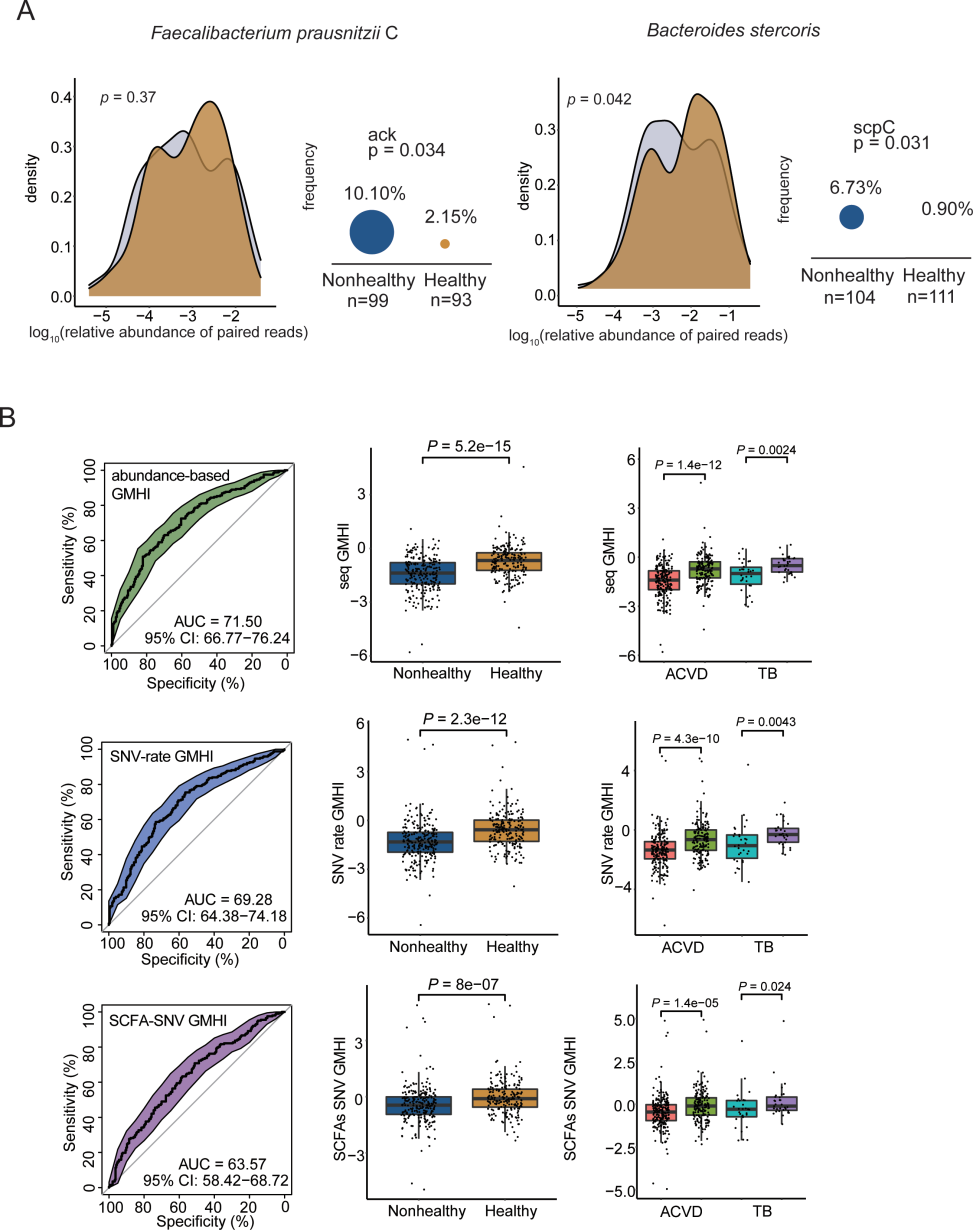
External validation of functional changes in SCFA production induced by SNVs and the predictive power of GMHIs. (**A**) Codon variant bias of two key SCFA-producing genes from two gut microbes (i.e., *ack* of *Faecalibacterium prausnitzii* C and scpC of *Bacteroides stercoris*) was validated using 446 additional metagenomic samples. The density plot shows the comparison of the sequence relative abundance of the two strains between healthy and nonhealthy groups using Wilcoxon rank-sum test. The variant ratios of *ack* and *scpC* genes were compared between host groups. *N* indicates the number of individuals with SNVs on each of these genes. When the prevalence was calculated, the denominator was *N*, and the numerator was the number of individuals having this codon variant bias. The *P* values from Fisher’s exact test and the significant difference were considered at a nominal level of *P* < 0.05. (**B**) The prediction performance of three GMHIs in the validation cohort. ROC analysis revealed that AUROC for three GMHI were 71.50%, 69.28%, and 63.57%, respectively. Each of these GMHIs can distinguish ACVD or TB from health. The GMHI values were compared between healthy and unhealthy groups with Wilcoxon rank-sum test. The significant difference was considered at a nominal level of *P* < 0.05. For all boxplots, the nonhealthy group was on the left and the healthy group was on the right.

To validate the above results, we further included three independent cohorts with a total of 446 samples, including 244 nonhealthy and 202 healthy individuals, related to atherosclerotic cardiovascular disease (ACVD) and tuberculosis (TB) (Supplementary file 2 and Table S9). The differences in relative abundance of the four strains changed compared with Fig. 5 due to the different cohorts (Fig. 7A). We observed that the relative abundances of *Faecalibacterium prausnitzii* C (*P* = 0.37) were no longer significantly higher in the healthy group than in the nonhealthy group, while the abundances of *Faecalibacterium prausnitzii* D (*P* < 0.001) and *Bacteroides stercoris* (*P* = 0.042) were higher in the healthy group. Interestingly, we still observed differential patterns of codon variant bias in *ack* gene of *Faecalibacterium prausnitzii* C (*P* = 0.034) and *scpC* gene of *Bacteroides stercoris* (*P* = 0.031); however, other codon biases have not been proven (Supplementary file 1 and Fig. S5). We speculated that the other three genes were not verified due to the low probability of codon variant bias (initiator and terminator), which can also be confirmed from Fig. 5. Overall, we demonstrated that SCFA metabolic genes on specific gut microbes exhibit codon variant bias (initiator and terminator) in hosts with different health states.

Next, the ACVD and TB cohorts were further validated for GMHI based on SNV profiles (Supplementary file 2 and Tables S10–S12). Overall, the three different GMHIs showed acceptable prediction accuracy, respectively. The validation accuracy (i.e., AUROC) of abundance-based GMHI was 71.50% (Wilcoxon rank-sum test, *P* = 5.2e−15), that of SNV-rate GMHI was 69.28% (Wilcoxon rank-sum test, *P* = 2.3e−12) and that of SCFA-SNV GMHI was 63.57% (Wilcoxon rank-sum test, *P* = 8e−7). Furthermore, these GMHI can distinguish ACVD or TB from health. Collectively, we demonstrated that health status changes can be distinguished by genetic changes in gut microbiome and highlighted the importance of SNV-induced functional changes related to SCFA production which underlie the pathogenesis of inflammatory bowel diseases and many diseases that were not interrogated before.

## DISCUSSION

The genetic composition of gut microbes was believed to be ever-changing for microbial adaptation under different host intestinal environments, where SNV is one of main representative forms (33–35). Here, we described for the first time the universal evolutionary patterns of gut microbes under different health status in multiple human cohorts (3). The previous study has provided insights that SNV makeup did not correlate with change in abundance (36) even in shallow sequencing data. Therefore, the population-level genetic processes can be a new information layer of microbiome data for predictive modeling of diseases. Next, the highly diverse evolutionary patterns have been found across microbial strains (5, 6, 11, 36), which highlighted the importance of in-depth understanding the relationship between the scale and speed of gut microbial evolution and host disease development.

Gut microbes can modulate the host gut health through SCFA production, which contributes to intestinal homeostasis and the regulation of energy metabolism (37). Certain major SCFA-producing bacteria in the gut are of concern due to their remarkable genetic or microbial evolutionary diversity difference between healthy and nonhealthy groups. *Lachnospiraceae* is a typical group of acetate and butyrate-producing bacteria, which provides beneficial effects for the host (38). In our study, more than half of the gut microbes with different SNV rates belong to the *Lachnospiraceae*. About 30% of the strains that are different in nucleotide diversity belong to *Bacteroides*, which are common producers of acetate and propionate. These results suggest that the evolution driven by intestinal selection pressure related to health status may play a role in the activity of SCFA-related genes. We next demonstrated that adaptive evolution affected the metabolic activity of SCFA genes under multiple medical conditions. The suppressed production of SCFAs can be related to the unfavorable variants that occurred in the related gene coding regions. We supposed that larger pN/pS may contribute to the tendency of codon variants, and variants on initiators and terminators would link to potential functional deficits in the gut microbiota leading to multiple chronic diseases. Interestingly, we did observe that a large number of codons tend to mutate to terminators in nonhealthy group compared with healthy group in four strains. Furthermore, this tendency does not occur in healthy groups, even if the genes of these microbes are subjected to greater intestinal selection pressure. Therefore, we infer that the intestinal selection pressure driven by different host health status promotes the obvious codon variant bias of SCFA gene. Collectively, we concluded that not only did the nonhealthy individual have fewer SCFA producers in the gut microbiota, but also these producers’ gene expression was loss of function due to adaptive variants.

Biomarker identification is one key goal toward the establishment of gut-microbiome-based prediction model for chronic diseases. On the one hand, it can be applied to non-invasive diagnosis, and on the other hand, it also provides a vision for constructing the causal relationship between host health and gut microbes. The composition of gut microbes has become the most basic biomarker, followed by functional genes, metabolite, and clinical characteristics (39, 40). Recently, we have tried to build diagnosis model based on SNVs or combined profiles and performed well (9, 11). GMHI was a powerful index to distinguish healthy from nonhealthy groups based on species relative abundance (33). We used GMHI based on SNV rate to compare healthy and nonhealthy individuals, and we demonstrated that the SNV rate was an independent indicator to evaluate the health status of hosts. At the SNV level of SCFA gene, it may help to understand the deep mechanism of the relationship between microbes and host health. At present, it may be difficult to achieve a unified model for assessing host health based on gut microbes, but the genetic evolution of gut microbes can provide additional insights.

A few limitations of our study should be noted. First, the sample size of our meta-analysis is not quite large, even we have included as many case samples as we can from the public database. In the discovery cohort, we included 1,711 samples of 12 phenotypes from 16 studies and while 446 samples were collected in the validation cohort. A few studies that also attempted to explore the universal gut microbial markers for multiple chronic diseases usually include over 3,000 metagenomic samples in the meta-analysis. In this big-data era, more metagenomic data should be included into meta-analysis to validate our findings related to gut microbial genetic variation affecting host health. Second, we supposed to construct an association between the SNV and more host phenotypes, including blood pressure, high-density lipoprotein, low-density lipoprotein, etc. However, our attempt was failed due to the lack of sample metadata. Accordingly, it is urgent to call for open-data science that lets scientists employ more publicly accessible data to explore the consensus SNV signatures associated with more host phenotypes (7).

## Data Availability

The datasets supporting the conclusions of this article are included within the article and its supplemental files (Tables 1 and S12), and no additional sequencing data are generated in this study. The script can be found at https://github.com/HNUmcc/Meta_SNV_2157, and the corresponding author (Jiachao Zhang) can be contacted for additional information.

## SUPPLEMENTAL MATERIAL

File S1 msystems.00828-23-s0001.docx Fig. S1 to S5.

File S2 msystems.00828-23-s0002.xlsx Tables S1 to S12.

ASM does not own the copyrights to Supplemental Material that may be linked to, or accessed through, an article. The authors have granted ASM a non-exclusive, world-wide license to publish the Supplemental Material files. Please contact the corresponding author directly for reuse.
