# Supplementary material for: Cross-cohort single-nucleotide-variant profiling of gut microbiota suggests a novel gut-health assessment approach
Source: mSystems. 2023 Oct 31;8(6):e00828-23. doi: 10.1128/msystems.00828-23 (PMC10734426; doi:10.1128/msystems.00828-23)
Supplement: File S1 — Fig. S1 to S5. [file msystems.00828-23-s0001.docx]

**
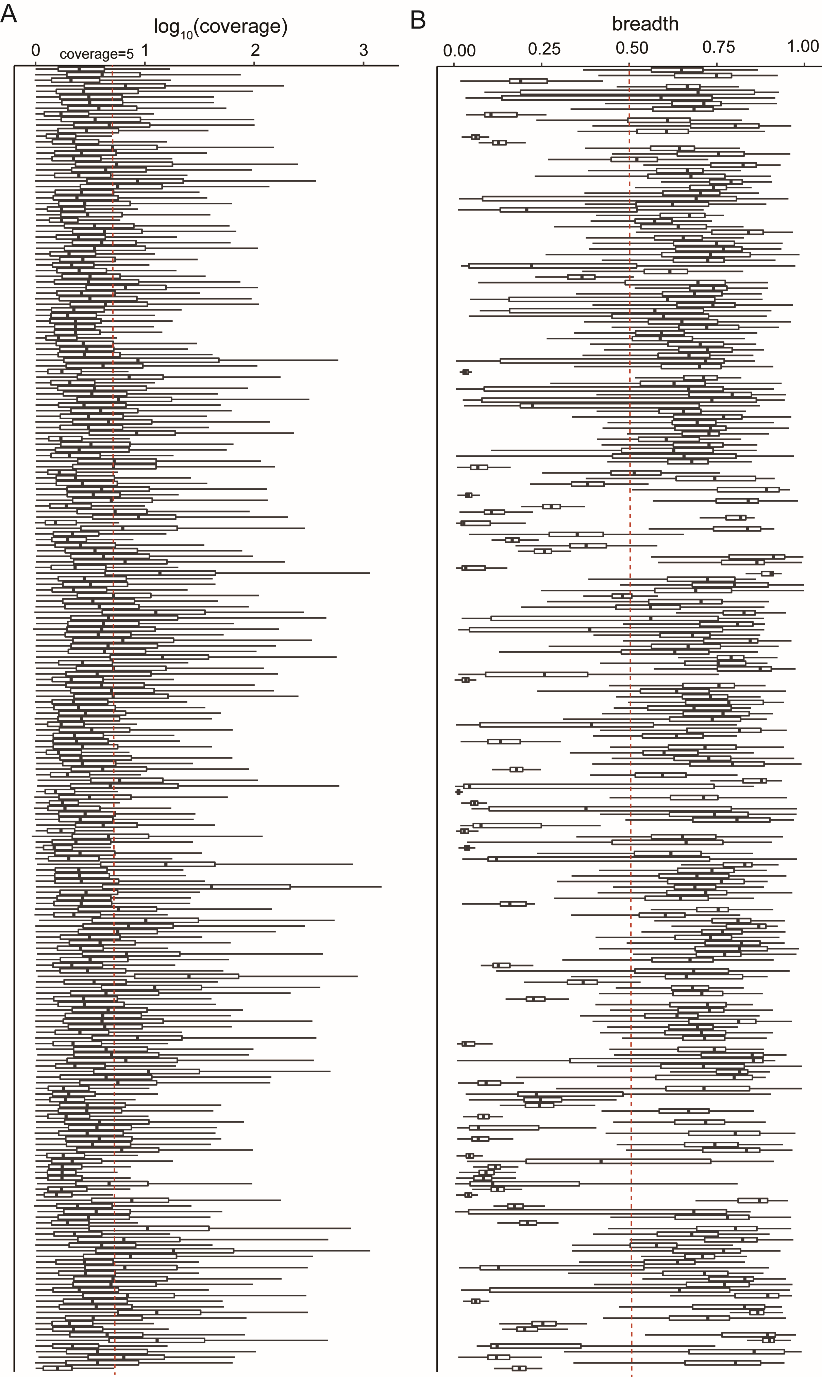
**

**Figure S1. Coverage and breadth profiles of the mutated genomes. A)** 233 mutated genomes in at least 10% of the samples were concerned, and their average coverage in the detected samples was evaluated. The coverage of SNV was required to be 5, and that's the minimum limit, which could cover most of the genomes. **B)** The breadth of the above samples was also evaluated and compared, and most strains have high breadth, which can meet the annotation of SNV.


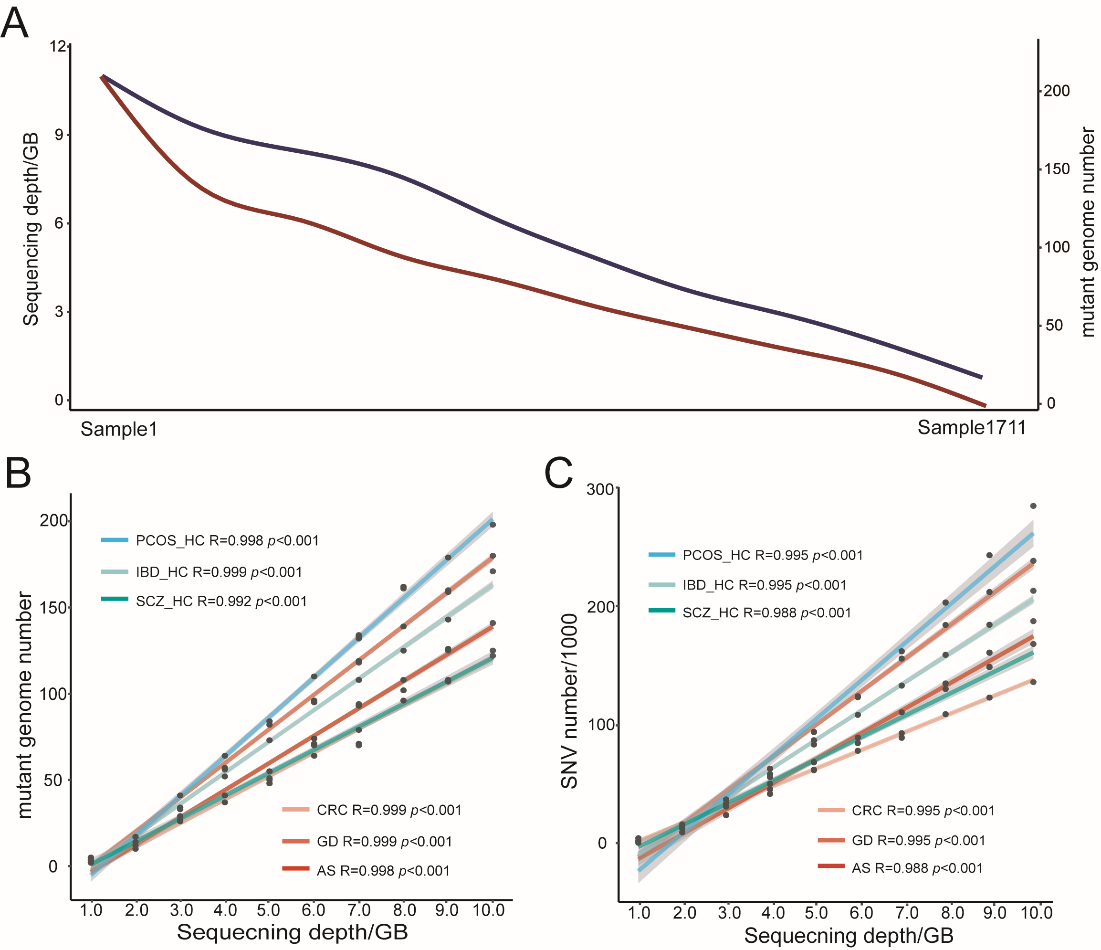


**Figure S2. Evaluation of the influence of sequencing depth by simulated data. A)** The decreasing trend of the sequencing depth and the number of mutated genomes of 1711 samples, and the cross section represents the same sample. The result showed that the number of mutated genomes is related to the sequencing depth. **B-C)** The simulated data showed the strong positive correlation in the number of mutated genomes and SNV and the sequencing depth of samples and the pipeline of simulated data can be found in the “Methods”.

**
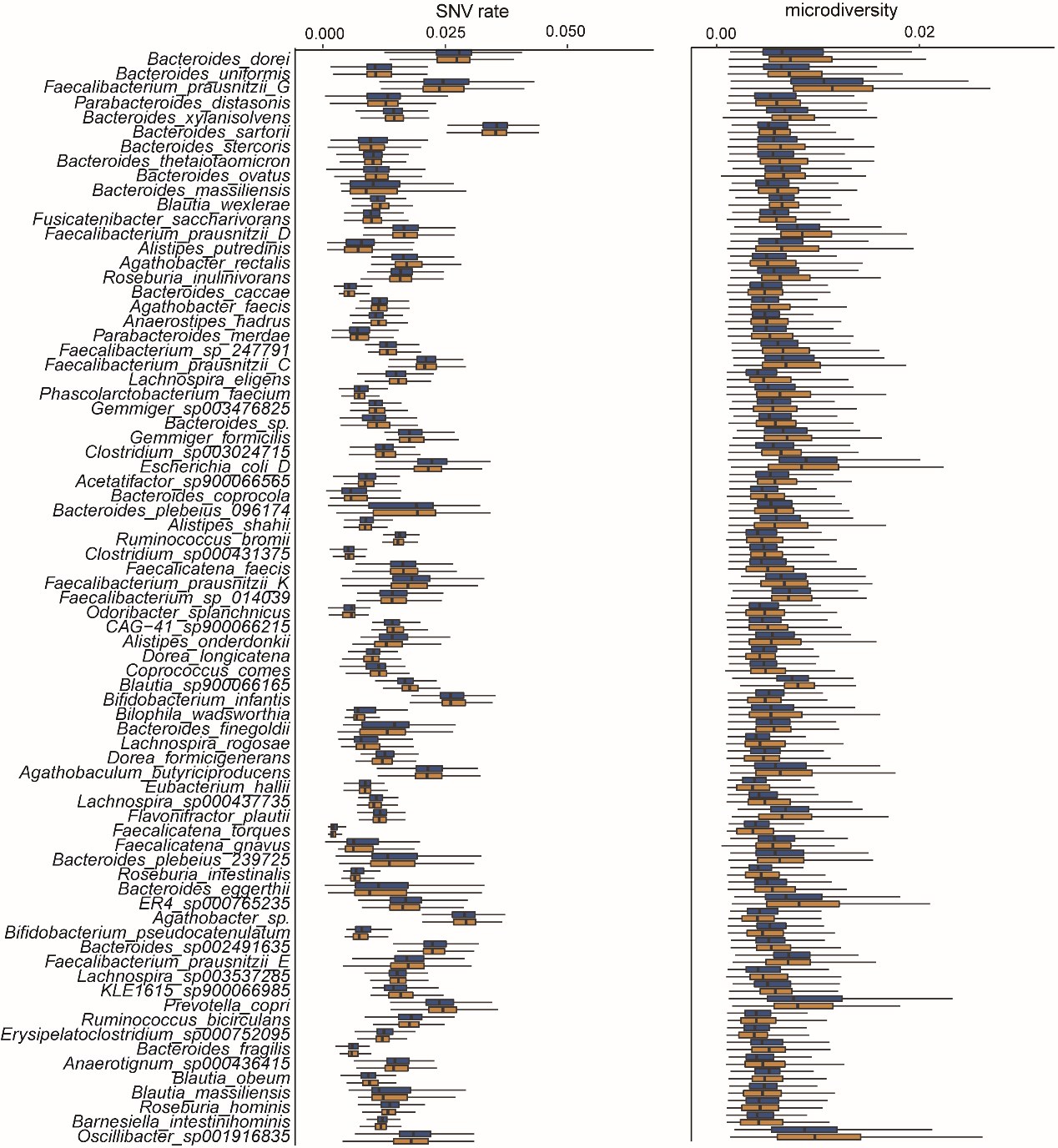
**

**Figure S3. SNV rate and nucleotide diversity of 75 strains.** The boxplot showed that the values of SNV rate and nucleotide diversity of 75 strains.

**
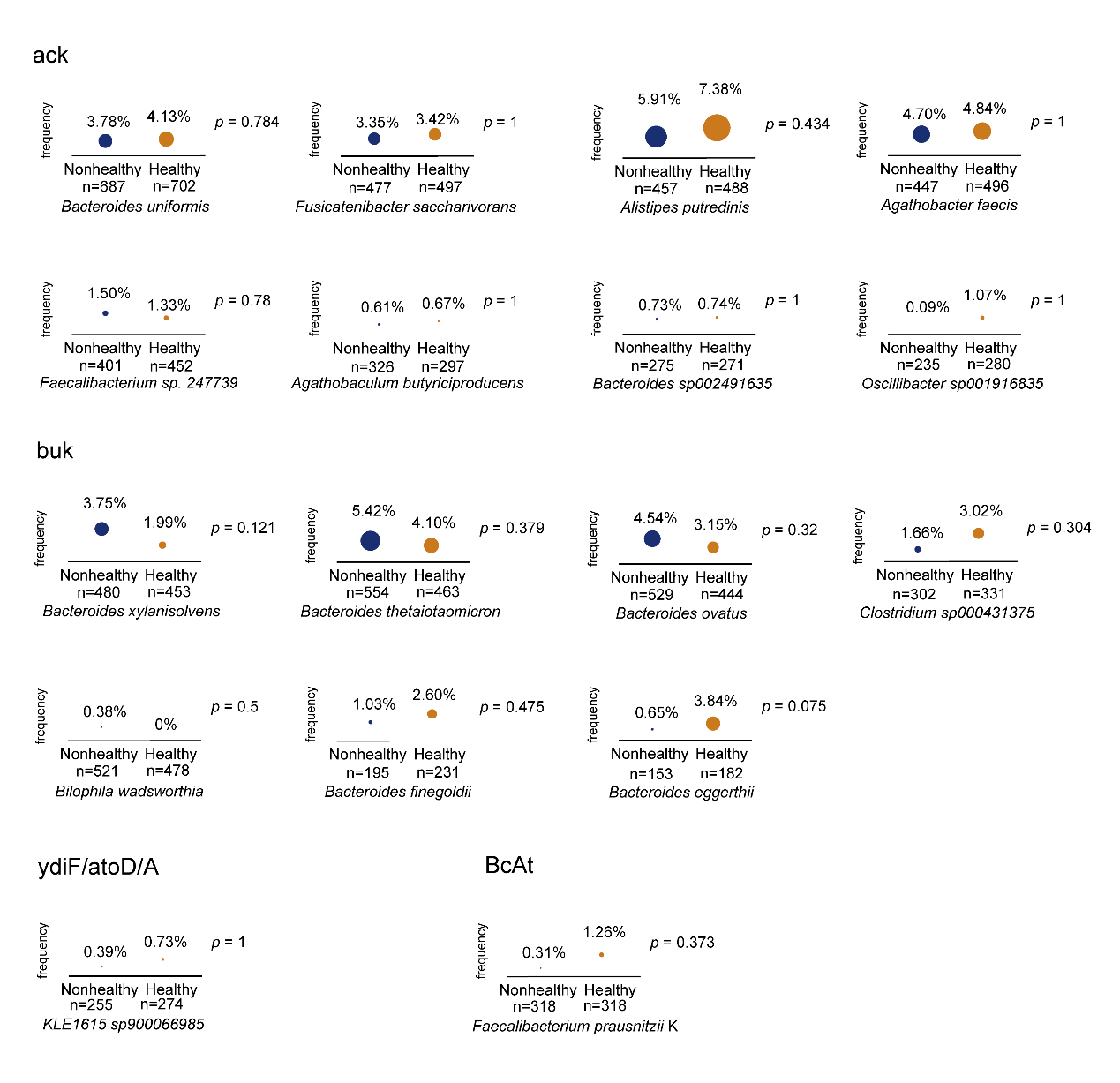
**

**Figure S4. Codon mutation bias in SCFAs gene on initiator and terminator.** All codon mutation bias of SCFAs gene of the strain marked with red font in Figure 3 was shown. N represented the number of individuals with SNV on these genes. When the prevalence was calculated, the denominator was N, and the numerator was the number of individuals with codon mutation bias. The *p* values were determined with the Fisher's exact test.


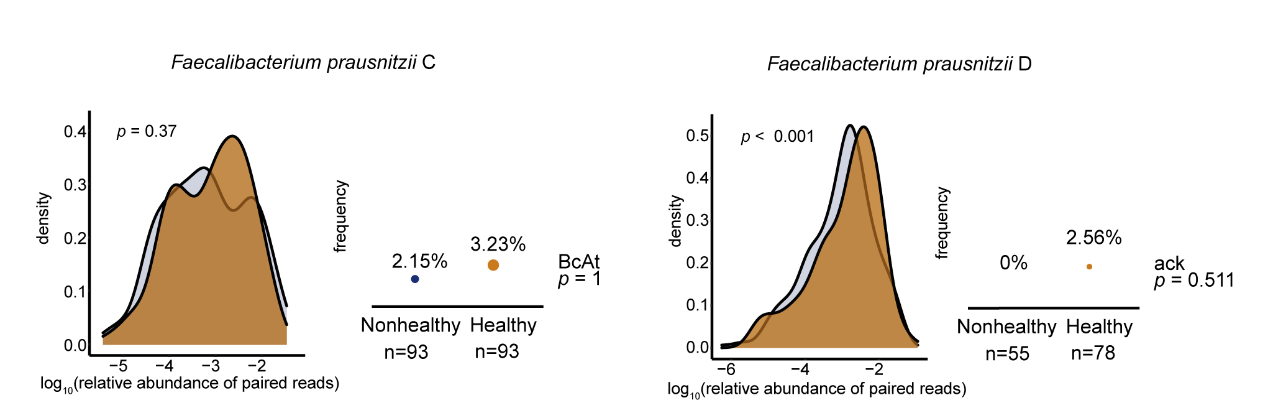


**Figure S5. Validation cohorts of codon mutation bias in SCFAs gene.** The abundance of three strains and the prevalence of characteristic codon mutation bias were described. Validation cohorts confirmed the characteristic codon mutation bias on ack gene of *Faecalibacterium prausnitzii* C and scpC gene of *Bacteroides stercoris*. However, BcAt gene of *Faecalibacterium prausnitzii* C (*p* = 1), ack gene of *Faecalibacterium prausnitzii* D (*p* = 0.511) have not been confirmed. The *p* values were determined with the Fisher's exact test.
